# Bleeding Risk in Post-bariatric Abdominoplasties: A Large Cohort Study

**DOI:** 10.1007/s00266-025-04887-9

**Published:** 2025-05-27

**Authors:** Nicole Garcia, Ishith Seth, Gianluca Marcaccini, Warren M. Rozen, Roberto Cuomo

**Affiliations:** 1 Department of Plastic and Reconstructive Surgery, Peninsula Health, Victoria, 3199 Australia; 2 Faculty of Medicine and Surgery, Peninsula Clinical School, Monash University, Melbourne, Australia; 3 Unit of Plastic and Reconstructive Surgery, Department of Medicine, Surgery and Neuroscience, University of Siena, Siena, Italy

**Keywords:** Post-bariatric abdominoplasty, Bleeding risk, Hypertension, Bariatric surgery, Thromboprophylaxis

## Abstract

**Background:**

Post-bariatric abdominoplasty is a standard procedure to improve body contour and quality of life following massive weight loss. However, perioperative bleeding remains a concern, particularly in the context of thromboprophylaxis and pre-existing comorbidities. This study investigates the risk factors for bleeding, including hypertension and bariatric surgery type, in patients undergoing abdominoplasty post-bariatric surgery.

**Methods:**

Patient data were retrospectively collected from a tertiary centre in Italy. Patient demographics, comorbidities, bariatric surgery type, and thromboprophylaxis use were analysed. Univariable and multivariable regression analyses identified significant predictors of bleeding. Risk ratios (RR) were calculated for bleeding outcomes based on bariatric surgery type and patient variables.

**Results:**

A total of 201 patients who underwent abdominoplasty following bariatric surgery were included in the analysis. The cohort had a median age of 44 years, with 71% female (142/201). Hypertension was significantly associated with bleeding risk (*β* = 0.1, *p* = 0.047; RR = 2.5, 95% CI: 1.02–6.01, *p* = 0.045). Among bariatric procedures, gastric banding conferred the highest bleeding risk compared to mini-gastric bypass (RR = 6.8, 95% CI: 2.09–21.8, *p* = 0.001). Sleeve gastrectomy also showed a higher bleeding risk than mini-gastric bypass (RR = 3.3, 95% CI: 1.24–8.5, *p* = 0.016).

**Conclusion:**

Hypertension emerged as an independent risk factor for bleeding in post-bariatric abdominoplasty, increasing risk by more than twofold. Additionally, bariatric surgery type influenced outcomes, with gastric banding and sleeve gastrectomy associated with higher bleeding risk compared to mini-gastric bypass. These findings highlight the importance of preoperative optimisation and surgical planning to mitigate bleeding complications.

**Level of Evidence V:**

This journal requires that authors assign a level of evidence to each article. For a full description of these Evidence-Based Medicine ratings, please refer to the Table of Contents or the online Instructions to Authors  www.springer.com/00266.

## Introduction

Abdominoplasty post-bariatric surgery is a frequently performed surgical procedure to improve quality of life after massive weight loss [1–3]. While generally considered safe and effective, it has risks, including venous thromboembolic events (VTE). To mitigate this risk, patients routinely receive thromboprophylaxis. Patients who undergo massive weight loss are predisposed to VTE due to prolonged immobility, elevated BMI, and post-bariatric physiological changes [4, 5]. As such, thromboprophylaxis is routinely administered to mitigate these risks, with evidence demonstrating a 50–70% reduction in VTE incidence across diverse surgical populations [6–9].

The administration of chemoprophylaxis, particularly in the perioperative period, introduces an opposing risk: increased bleeding complications. Post-bariatric patients have unique vulnerabilities, including altered vascular anatomy, nutritional deficiencies, and changes in haemostatic balance, which may exacerbate bleeding tendencies [10–12]. Several studies have highlighted increased hematoma and seroma formation following body contouring surgery in this cohort, raising concerns regarding the optimal balance between thromboprophylaxis and perioperative safety [13, 14]. For instance, in a recent single-centre study of 102 patients [10], although a higher risk of bleeding was observed in those with significant weight loss, the use of prophylactic low-molecular-weight heparin showed only a borderline effect (*p* = 0.06) on the incidence of hematomas requiring surgical intervention. This suggests that chemoprophylaxis is not the principal driver of postoperative bleeding, but larger cohorts are needed to confirm these findings and refine clinical protocols.

Given the complex interplay between thrombosis prevention and bleeding risk in post-bariatric abdominoplasty patients, further investigation is warranted to identify modifiable patient factors contributing to bleeding complications. Specifically, understanding demographic, procedural, and prophylactic variables will aid in optimising perioperative care. This study aims to analyse predictors of bleeding in post-bariatric abdominoplasty patients, evaluate whether these risks can be mitigated, and determine the appropriate balance between VTE prevention and minimising perioperative bleeding complications.

## Methodology

This retrospective cohort study was conducted at the University of Siena Teaching Hospital, a tertiary referral centre in Italy. Patient data were collected between January 2020 and December 2022, with postoperative follow-up assessments performed at 1 month, 3 months, 6 months, and up to 12 months. Institutional ethics committee approval was obtained, and the study was classified as low-risk/negligible-risk research.

### Study Setting and Patient Selection

All abdominoplasties were performed by board-certified consultant-level surgeons with over five years of independent practice experience. The bariatric surgeries had been carried out by a separate, specialised Bariatric Surgery Unit (2–3 years prior to body contouring), ensuring no overlap in surgical teams.

Eligible patients included those aged 18 years or older who underwent abdominoplasty following bariatric surgery and had stable weight for at least three months before surgery. Patients were excluded if they underwent dermatolipectomy without upper flap dissection, partial abdominoplasty, or revision procedures. Other exclusion criteria included pre-existing coagulopathies, hematological disorders (e.g., Klinefelter syndrome), vitamin K deficiencies, incomplete medical records, or loss of follow-up within the 12-month postoperative period.

### Surgical Technique

All abdominoplasties were performed by four board-certified plastic surgeons (each with over five years of experience). A low transverse incision was made, and a traditional “full undermining” technique was adopted in all cases, elevating the abdominal flap off the rectus fascia up to the costal margin. Rectus diastasis was repaired when indicated, and liposuction was performed in selected patients to improve contour. We did not conduct a surgeon-specific or technique-specific subgroup analysis because it was not the primary aim of this study.

### Surgical Adjuncts: Diastasis Correction and Liposuction

In patients presenting with symptomatic or clinically significant rectus diastasis, we performed a standard plication of the anterior rectus sheath using interrupted or running sutures, according to surgeon preference. The exact measurement of diastasis width was not systematically documented and therefore was not included in the quantitative analysis.

Traditional suction-assisted liposuction (SAL) using standard cannulas was performed in selected patients to refine the abdominal and flank contour. No power-assisted or ultrasound-assisted liposuction devices were employed in our cohort. We did not routinely document the aspirate volume or the specific extent of liposuction, and thus, we were unable to perform a formal correlation between liposuction volume and bleeding outcomes.

Data were retrospectively extracted from the hospital’s electronic medical record system. The collected variables included patient demographics (age, sex, BMI, weight loss, smoking history), comorbidities (hypertension, diabetes), bariatric surgery details (type of procedure: gastric banding, sleeve gastrectomy, or mini-gastric bypass), and perioperative management. Bleeding events were defined as clinically significant intraoperative bleeding requiring blood transfusion, postoperative hematoma requiring surgical evacuation or needle aspiration, or a drop in haemoglobin exceeding 2 g/dL within 48 h postoperatively. Although intraoperative bleeding severe enough to require transfusion is indeed extremely rare, we included it to ensure all clinically significant events were captured. A detailed review of these rare cases revealed no direct correlation with surgical technique errors; instead, they were attributed to patient-specific factors. such as coagulopathy, uncontrolled hypertension, or anatomic vascular variations.

All patients were assessed preoperatively for venous thromboembolism (VTE) risk according to our institutional guidelines, considering factors, such as body mass index, personal or familial history of thrombosis, and obesity-related comorbidities (e.g., hypertension, diabetes). Patients at higher risk received mechanical and chemical prophylaxis, while lower-risk patients were managed with mechanical measures alone. Mechanical methods (graduated compression stockings and/or intermittent pneumatic compression devices) were employed for every patient intraoperatively and continued until full ambulation. Of the 201 patients, 59 (29%) were administered subcutaneous enoxaparin, reflecting those identified with additional risk factors. Among these 59 patients, 21 (10% of all patients) were given a prophylactic dose of 20 mg once daily, whereas 38 (19%) received ≥ 40 mg (e.g., 40 mg once or twice daily), at the attending surgeon’s discretion. The initial enoxaparin dose was typically administered 6–8 h postoperatively. The remaining 142 patients (71%) received no chemical prophylaxis and were managed exclusively with mechanical measures. Detailed dosage records, including timing and subsequent doses, were retrieved from the hospital’s electronic medical record system.

In this study, we defined a postoperative bleeding event as one meeting at least one of the following criteria: a clinically evident hematoma requiring needle aspiration or surgical evacuation, a drop in haemoglobin of ≥ 2 g/dL within the first 48 h, or the necessity for blood transfusion or haemodynamic support. Bleeding episodes were further classified as early if they occurred within 48 hours of surgery or late if they manifested after postoperative day 2.

The primary outcome measure was the incidence of bleeding events within 30 days postoperatively. Secondary outcomes included the association of bleeding with patient-specific factors, perioperative management, and the type of bariatric surgery performed before abdominoplasty.

### Statistical Analysis

Generalised linear regression was utilised to analyse nonparametric independent variables in the univariate data analysis. Following this, multivariable analysis was performed on a limited number of patient variables to investigate variables that may influence each other. Multivariable analysis was also performed, specifically on the types of bariatric surgery done before abdominoplasty. Risk ratios were calculated for all variables and the type of bariatric surgery the patients received before their abdominoplasty. Statistical package STATA (v16, StataCorp, Texas, USA) was used for data analysis.

## Results

Our cohort consisted of 201 patients who underwent an abdominoplasty following bariatric surgery. The demographics of our sample are presented in Table 1. Patients had a median age of 44 years at the time of the abdominoplasty, with the majority of the cohort being female (142/201, 71%). Patient comorbidities of preoperative, controlled hypertension and diabetes were noted, with 21% (42/201) and 7% (14/201) of the cohort having these, respectively. Body mass index (BMI) at the time of abdominoplasty was also recorded, with a cohort median of 28 kg/m2. In addition, the median weight loss at the time of abdominoplasty was 47 kg. Almost a third of patients received perioperative VTE prophylaxis (59/201, 29%). Nearly a third of patients received perioperative VTE prophylaxis (59/201, 29%). Of these 59, 21 (36%) received 20 mg once daily, while 38 (64%) received ≥ 40 mg daily. No patients developed heparin-induced thrombocytopenia or other significant adverse effects attributable to enoxaparin. Postoperative bleeding events were noted in 9% of patients (18/201).

**Table 1:** Patients and surgical demographics

Median age in years (IQR)	44 (19)
Male (%)	59/201 (29)
Patients with hypertension (%)	42/201 (21%)
Patients with diabetes (%)	14/201 (7%)
Patients who smoke (%)	37/201 (18%)
Median BMI in kg/m^2^ (IQR)	28 (4)
Median weight loss in kg (IQR)	47 (24)
Bariatric surgery type	
Gastric banding	9/201 (4%)
Mini-gastric bypass	142/201 (71%)
Sleeve gastrectomy	50/201 (25%)
Patients who received enoxaparin	59/201 (29%)
Patients who experienced bleeding events	18?201 (9%)

Of the 201 patients in this study, 18 (9%) experienced a postoperative bleeding event according to the abovementioned criteria. In total, 14 (78%) bleeding episodes were classified as early (within 48 h of surgery) and 4 (22%) were late (detected between postoperative days 3 and 5). Nine patients (4.5%) required surgical evacuation of a hematoma, whereas six (3%) were managed successfully with ultrasound-guided needle aspiration. In three patients (1.5%), the bleeding was identified primarily through a drop in haemoglobin of ≥ 2 g/dL but did not necessitate any invasive procedure. Two patients received intraoperative blood transfusions due to haemoglobin levels falling below 8 g/dL in conjunction with haemodynamic instability. All bleeding complications were addressed promptly, and no additional hematomas or significant bleeding events were noted beyond the fifth postoperative day. No patients experienced irreversible morbidity or mortality related to these bleeding complications. Before their abdominoplasty, patients received one of three types of bariatric surgery: gastric band, mini-gastric bypass, or sleeve gastrectomy. Most patients underwent a mini-gastric bypass (142/201, 71%), and a quarter of patients underwent a sleeve gastrectomy (50/201, 25%).

Of all the variables interrogated, only the comorbidity of preoperative, controlled hypertension demonstrated a statistically significant association with bleeding risk when performing a univariable regression analysis (*β* = 0.1, *p* = 0.047) (see Table 2). A pair-wise comparison of the bariatric surgery type demonstrated a statistically significant negative association of mini-bypass and sleeve gastrectomy when compared directly with gastric banding (*β* = −0.27, *p* = 0.006 and *β* = −0.13, *p* = 0.007, respectively). When selecting variables that have previously been demonstrated to relate to an increased postoperative bleeding risk (hypertension, diabetes, smoking status, BMI and whether or not patients received chemical VTE prophylaxis), only the presence of pre-morbid hypertension demonstrated a statistically significant association with bleeding risk (*β* = 0.1, *p* = 0.04) (see Table 3).

**Table 2:** Univariable regression analysis of patient and surgical demographics

Patients variable	Relationship coefficient	*p* value	95% CI
Age	0.002	0.14	−0.001, 0.01
Male sex	−0.007	0.88	−0.09, 0.08
Hypertension	0.1	0.047	0.01, 0.19
Diabetes	−0.1	0.22	−0.25, 0.06
Smoker	0.06	0.29	−0.05, 0.10
BMI	−0.003	0.72	−0.02, 0.01
Weight loss	0.001	1	−0.003, 0.003
Bariatric surgery type	0.03	0.51	−0.05, 0.10
Mini-gastric bypass vs. Gastric banding	−0.27	0.006	0.076, 0.46
Sleeve gastrectomy vs. Gastric banding	−0.013	0.007	0.03, 0.22
Enoxaparin	0.04	0.35	−0.05, 0.13

**Table 3:** Multi variable regression analysis

Patients variable	Relationship coefficient	*p* value	95% CI
Hypertension	0.1	0.04	0.006, 0.2
Diabetes	−0.08	0.3	−0.23, 0.08
Smoker	0.08	0.15	−0.03, 0.18
BMI	−0.004	0.64	−0.02, 0.1
Enoxaparin	0.04	0.35	−0.04, 0.13

Patient variables selected for multivariate analysis were performed based on previous literature

Risk estimation was performed regarding the types of bariatric surgery patients received before their abdominoplasty. Gastric banding had a significant, almost sevenfold increased risk of bleeding compared to mini-bypass (RR = 6.8, *p* = 0.001). Sleeve gastrectomy had an important, more than threefold increased risk of bleeding when compared to mini-bypass (RR = 6.8, *p* = 0.016). Sleeve gastrectomy had a twofold increased risk of bleeding when compared directly with gastric banding; however, this was not statistically significant (*p* = 0.2) (see Table 4).

**Table 4:** Risk estimation of types of bariatric surgery

Bariatric surgery type	Risk ratio	*p* value	95% CI
Gastric banding vs. Mini-gastric bypass	6.8	0.001	2.09, 21.8
Sleeve gastrectomy vs. Mini-gastric bypass	3.3	0.0016	1.24, 8.5
Sleevegastrectomy vs. Gastric banding	2.1	0.2	0.68, 6.4

Risk analysis was also performed on the patient factors as outlined in Table 5. This demonstrated a significant, more than twofold increased risk of bleeding in patients who had preoperative hypertension before their abdominoplasty, irrespective of their type of bariatric surgery (*p* = 0.045). Smoking pre-abdominoplasty and receiving chemical VTE prophylaxis also demonstrated an increased risk of bleeding following their body contouring procedure, however, these were not statistically significant (RR = 2.01, *p* = 0.15 and RR = 1.5, *p* = 0.4, respectively).

**Table 5:** Risk estimation of patient variables

Patient variable	Risk ratio	*p* value	95% CI
Hypertension	2.5	0.0045	1.02, 6.01
Smoker	2.0.1	0.15	0.78, 5.2
BMI	0.94	0.53	0.78, 1.13
Enoxaparin	1.5	0.4	0.6, 3.56

Diabetes could not be analysed due to the collinearity of the data

## Discussion

This study analyses risk factors associated with bleeding in patients undergoing abdominoplasty after bariatric surgery. It interrogates individual patient factors and procedural details. Although numerous patient factors were investigated, only preoperative hypertension demonstrated a statistically significant association with bleeding risk. In our cohort, gastric banding showed a higher risk of bleeding compared to sleeve gastrectomy and mini-bypass. However, only 9 of the 201 patients (4.5%) had undergone a gastric band procedure, whereas 142 (71%) had a mini-bypass and 50 (25%) had a sleeve gastrectomy. This small sample size likely magnifies the observed difference in bleeding rates. Moreover, gastric banding was more commonly performed in earlier years, reflecting older patient selection protocols. Patients with a band may also experience less remission of obesity-related comorbidities, such as hypertension, than those with malabsorptive or mixed bariatric procedures, and residual hypertension could potentially increase bleeding risk. Future studies including larger, more balanced samples of each bariatric surgery type will help clarify whether the elevated risk seen with banding persists when patient numbers are more comparable.

MA multivariable analysis reinforced the association of preoperative hypertension at the time of abdominoplasty to an increased risk of bleeding; however, all other outcomes investigated demonstrated weak relationships with bleeding outcomes. Despite this, when comparing hypertensive and non-hypertensive groups directly, the risk ratio for patients with recorded preoperative hypertension was elevated at 2.5. This underscores that hypertension is an independent risk factor for post-abdominoplasty bleeding, carrying a more than twofold risk of doing so. Hypertension has previously been demonstrated as a risk factor for haematoma formation, a secondary measure of postoperative bleeding, in patients who have undergone an array of reconstructive procedures, including panniculectomies [17–20] as well as head and neck reconstruction. It has been previously demonstrated that a high BMI increases the average diameter of blood vessels and that subsequent loss of body weight does not reduce this size [21, 22]. Resultantly, it may be logically extrapolated that in our cohort, hypertension coupled with larger than standard blood vessels would thus impart a higher bleeding risk [21, 22].

Interestingly, although prior studies link elevated BMI to higher hematoma rates, our analysis showed only minimal significance. One possible explanation is the relatively narrow range of BMIs in our post-bariatric population, as most patients had already experienced substantial weight loss, thereby limiting the direct impact of BMI on surgical bleeding risk [23–26].

Studies with similar cohorts also looked into patient factors in those who underwent abdominal body contouring surgery post-bariatric surgery; however, hypertension was only related to a non-significant increased incidence of patients who underwent abdominal body contouring surgery without a history of prior bariatric surgery [10, 19, 24].

Previous studies have demonstrated high preoperative BMI, diabetes mellitus, increased age and smoking to be independent significant risk factors for general complications in post-bariatric abdominoplasty patients [15–17]. Overweight and obese patients, according to BMI, have demonstrated an increased incidence of haematoma when undergoing aesthetic procedures. Preoperative weight has also been correlated with haematoma or bleeding following abdominoplasty. Further, a large cohort study using a nationwide database (n > 20,000) demonstrated that diabetes is an independent risk factor for receiving a blood transfusion post-abdominoplasty in the post-bariatric cohort. Total weight loss, high BMI before abdominoplasty, or having diabetes were not related to any bleeding events in our study. This will likely reflect our cohort size and overall patient sample and may result from an utterly standardised approach and technique to abdominoplasty inherent to a single-centre study.

Enoxaparin administration did not reveal an increased incidence or risk of perioperative bleeding in our study, despite previously having been demonstrated to be associated with higher bleeding rates in body contouring patients. This may also reflect our cohort, specifically the interplay of patient factors not accounted for in our study, such as a history of coagulation disorders that may account for an increased or decreased risk of perioperative bleeding [27–30]. Nevertheless, this is the first study to examine patient factors and bleeding risks in a cohort of body-contouring patients, specifically post-bariatric surgery.

A novel finding of our study was the relationship between the type of bariatric surgery before abdominoplasty and the bleeding risk this conferred. Both gastric banding and sleeve gastrectomies before an abdominoplasty impart a significantly higher risk of bleeding when compared to patients who underwent a mini-gastric bypass as their bariatric surgery. Those who underwent sleeve gastrectomy also had a twofold risk of bleeding compared to those who underwent gastric banding, though this finding did not reach significance. Interestingly, without taking a subsequent abdominoplasty or body contouring surgery into consideration, gastric banding confers the lowest bleeding risk, followed by sleeve gastrectomy, with mini-gastric bypass having the highest bleeding risk. Whether or not these bariatric procedures were performed open or laparoscopically and how this could have affected subsequent operations in the same anatomical region were not noted in our study. It is also important to note that these are two independent procedures generally performed by two different surgeons from two different specialties, therefore conferring independent risks altogether.

Regarding the different bariatric procedures, at our institution, gastric banding was historically performed more frequently in earlier years, often in patients with a lower initial BMI or specific comorbidity profiles. More recently, mini-bypass and sleeve gastrectomy have become the predominant choices. We hypothesise that banding patients may not achieve the same degree of metabolic improvement (e.g., resolution of hypertension) as those undergoing malabsorptive or mixed procedures, potentially explaining their higher bleeding rates. However, we cannot definitively prove that the overall health status of banding patients was worse since we lack specific granular baseline data (e.g., precise comorbidity scores). A larger prospective analysis is required to determine whether these findings persist when controlling for temporal trends and patient selection criteria.

With this in mind, however, gastric banding is the least effective for resolving obesity-related comorbidities such as diabetes and hypertension due to its restrictive nature. It does not affect hormonal pathways like a malabsorptive bariatric surgery such as a mini-gastric bypass. As a result, the increased risk of bleeding in this subset of patients in our study may be related to their overall health and comorbidity profile, including the independent risk factor for bleeding, hypertension. As intimated above, additional factors to consider would be the procedural complexity of the abdominoplasty in a cohort of patients who have already had a minimum of one operation (as one bariatric surgery can be a bridge to another) in the same field and the inherent differences in patient selection criteria across bariatric surgery types that we are unable to account for.

Our study concludes that hypertension is an independent risk factor for increased bleeding in patients who have undergone an abdominoplasty following bariatric surgery and that the type of bariatric surgery prior affects your bleeding risk in a subsequent abdominoplasty. Our study has various strengths, including the number of patients included and the factors interrogated. This is the first study to review factors related to bleeding risk, specifically in a cohort of post-bariatric surgery patients. Furthermore, it is the first to review the bleeding risk and its relation to the type of bariatric surgery performed prior.

This study has several limitations that must be acknowledged. First, as a single-centre retrospective analysis, the findings are subject to inherent selection and reporting biases, which may influence the observed outcomes. Additionally, the sample size, while robust, remains limited for subgroup analyses, particularly when examining the impact of individual bariatric surgery types and patient-specific factors. A larger, multicenter cohort would enhance the generalizability of these findings and provide sufficient statistical power to explore less common variables and interactions further. Another limitation is the lack of granular data on intraoperative factors, such as blood loss quantification, duration of surgery, and detailed perioperative haemodynamic parameters, which may influence bleeding risk. Future prospective studies should incorporate standardised bleeding definitions, real-time intraoperative blood loss measurements, and rigorous follow-up protocols to ensure comprehensive data capture. Moreover, while this study identified an association between bariatric surgery types and bleeding risk, the underlying mechanisms remain unclear. Future investigations should include comparative histological analyses, examining vascular remodelling and tissue changes in post-bariatric patients to better elucidate the pathophysiology behind the increased bleeding tendencies observed in specific bariatric subgroups.

Another limitation involves the lack of comprehensive intraoperative and postoperative haemodynamic data. Although blood pressure is routinely monitored, our research database did not systematically capture these values. Consequently, we could not assess potential fluctuations in perfusion (e.g., low intraoperative versus higher postoperative blood pressure), which might increase bleeding risk in hypertensive patients. Future prospective data collection protocols should incorporate standardised blood pressure management recordings, surgery duration, and exact blood loss measurements to clarify their impact on postoperative bleeding events.

To address the research gap, future studies should include multicenter prospective cohorts to validate these findings across diverse populations and surgical settings. Randomised controlled trials comparing thromboprophylaxis protocols and their bleeding risk profiles in post-bariatric abdominoplasty patients also help define optimal perioperative management strategies. Additionally, studies integrating predictive modelling, such as machine learning algorithms, could assist in identifying high-risk patients and personalising perioperative care to mitigate bleeding risk while preventing thromboembolic events.

## Conclusion

This study highlights preoperative hypertension and bariatric surgery type as significant predictors of bleeding risk in post-bariatric abdominoplasty patients. Hypertension was identified as an independent risk factor, doubling the likelihood of bleeding complications and emphasising the need for thorough preoperative optimisation and perioperative monitoring. Additionally, gastric banding and sleeve gastrectomy were associated with a significantly higher bleeding risk compared to mini-gastric bypass, suggesting that surgical complexity and vascular changes following bariatric procedures influence outcomes. Our findings underscore the importance of individualised risk stratification and tailored perioperative strategies to improve patient safety. Future multicenter prospective studies with standardised bleeding definitions and mechanical analyses of vascular changes are needed to validate these results.

## Declarations

### Informed Consents

For this type of study informed consent is not required.
